# “Right-to-Try” experimental drugs: an overview

**DOI:** 10.1186/s12967-020-02427-4

**Published:** 2020-06-23

**Authors:** Vijay Mahant

**Affiliations:** MediLite Diagnostika, Inc, 12364 Carmel Country Rd, San Diego, CA 92130 USA

**Keywords:** “Right-to-Try” experimental drugs, Precision medicine, EHR, Artificial intelligence, Machine learning, Panomics, RWD, RWE, caTrip and caBIG

## Abstract

The “Right-to-Try” experimental drugs act passed by Donald Trump in 2018 provides an opportunity of early access to experimental drugs for the treatment of life-threatening diseases and a potential boon to many young and under-capitalized biotechnology or pharmaceutical companies. The pros and cons of experimental drugs, including a number of “cutting edge” scientific, clinical, and a number of synergistic approaches such as artificial intelligence, machine learning, big data, data refineries, electronic health records, data driven clinical decisions and risk mitigation are reviewed.

## Background

Over a century ago, most medications were the “wild west” with alleged therapeutic benefits. Most such medications sold to the public offered negligible or no evidence-based therapeutic efficacy and safety to the patient at large, except the “placebo benefit”—and hope—at the best. The drugs were unregulated and sold to the public in the USA and many parts of the world. To protect the public against the sale of misbranded, mislabeled and/or adulterated foods and drugs, the US in 1906 enacted [1] the “Pure Food and Drug Act” and this resulted in the establishment of the federal agency, the Food and Drug Administration (FDA).

## Historical overview

The “Right-to-Try” experimental drugs, however, originated [2–4] from the Abigail Alliance for Better Access to Developmental Drugs versus von Eschenbach. Due to Abigail’s high expression of EGFR, her oncologist recommended Abigail for her terminal head and neck cancer to try an investigational EGFR-targeted drug, C222 (Erbitux), which was then undergoing clinical trial for the treatment of colorectal cancer. Due to her ineligibility to participate in the clinical trials and denial by the FDA, Abigail’s father, Frank Burroughs sued the FDA in 2003 for access to the experimental drug, Erbitux, on the pretext that an investigational drug by terminally ill patients after phase I approval was a constitutional right. Abigail’s tragic story was one of the primary precursors and a “catalyst” that inspired patients and non-patients, including advocacy groups for access to unapproved therapies by the FDA.

In May 2018, President Donald Trump signed the “Right-to-Try” Act [5]. The legislation overcame many of the regulatory barriers, limited the risks to the sponsor while implementation of the act inherently burdened the sponsor. The “Right-to-Try” legislation is in essence a derivative of the Expanded Access Programs (EAPs). Advocates such as patients, families, friends and advocacy groups of the “Right-to-Try” legislation argue that the legislation is in line within the pre-existing framework of EAPs and that the legislation: (i) provides a “streamlined” avenue for making eligible drugs available to eligible patients with no other options; (ii) it increases patient’s engagement; (iii) it is a patient’s journey of self-actualization; (iv) it empowers the patient about his or her own health, well-being and quality of life; (v) it provides optimism and access to novel interventions with potential therapeutic benefits that may prolong life and improve quality of life; and (vi) the patient can be treated in the USA with valuable family time, more comfort and fewer risks than being treated overseas. The critics, on the other hand, argue: (i) there is an inherent safety risk that may potentially cause more harm to the patient or even death than the benefit because the experimental drug did not undergo rigorous testing; (ii) there is a lack of oversight by the FDA, except posting of the consolidated annual summary report; (iii) the patient in most cases has limited understanding of the informed consent due to complexity and confusion of the medical terminology used in the consent form; (iv) there are therapeutic misconceptions combined with high expectations and optimism by the patient; (v) there is potentially a considerable financial burden by the patient or the patient’s family because payors currently do not provide coverage and deny hospice care; (vi) there is a potential loss of trust in the regulatory agency, the sponsor and the health care provider; and (vii) there is a liability “immunity” for the health-care provider, including the drug sponsor for potential negative outcomes of the treatment unless the medical provider and the sponsor were engaged in “gross negligence, reckless or “wilful misconduct.”

Some of the major inherent limitations and often overlooked about the “Right-to-Try” experimental drugs are: (i) patient’s vulnerability due to lack of oversight by the FDA; (ii) there is a lack of clinical study protocol, including the lack of sufficient statistical power to detect the intended effect(s); (iii) the information is collected in a “piece-meal”; and (iv) a lack of systematic reporting about efficacy and the safety of the experimental drug that may potentially result in limited information for the health and safety of the public. Some of these issues may be addressed and resolved by utilizing EHR systems. EHR systems are maintained by health care providers and health care organizations for delivering patient care. EHR systems can thus easily lend themselves for integrating real-time electronic health care information about the patient across multiple health care providers.

One of the most important considerations and occasionally overlooked is comorbidity; it is quite common among cancer patients and it can potentially affect the treatment outcome, worsen the adverse effects due to polypharmacy and may even shorten the life. The prognosis of patients with comorbidity is often poor survival combined with poor quality of life and higher financial costs. The use of experimental drugs in patients with morbidity may thus be risky or limited unless the experimental drug has gone through further rigorous testing and/or the experimental drug is paired synergistically with an FDA-approved drug and appropriate tools are employed for monitoring efficacy and safety of the therapy. Treatment of metastatic cancer patients with comorbidity using experimental drugs would be even more challenging and riskier with many implications that may warrant further considerations that are in the best interest of the patient’s health, well-being and life, including additional financial burden.

## The “landscape, the ecosystem and the dynamics”

The implications of ethics, law, regulations, government policies, constitutional rights by terminal ill patients, patient advocacy groups, including stakeholders about the pros and cons echoed through the “ecosystem” of early access to investigational drugs. Under the “Right-to-Try” legislation, the eligibility to participate include: (i) the patient must have been diagnosed with a debilitating or life-threatening disease; (ii) the patient must have failed all standard of care treatments; (iii) the experimental or the investigational drug must have completed at least phase 1 trial; (iv) the patient must have signed an informed consent [6]; and (vi) the pharmaceutical company must be able to provide the experimental drug to the patient. Due to the potentially negative outcome about the therapeutic efficacy combined with the safety issues, most sponsors developing medications for life-threatening diseases have had reservations about participating in expanded access or the “Right-to-Try” programs. To de-risk the potential negative outcomes and the implications combined with an opportunity to target a much larger number of patients than to a few eligible patients under the early access programs, the sponsors’ primary goal has been to have full FDA approval of the drug. The drug approval process, of course, is lengthy and highly risky due to potential clinical trial failures along each step of the approval process. It is an expensive process—currently, estimated to be between US$ 1.9–2.5 billion [7, 8].

On the other hand, China for almost two decades approved a number of experimental drugs. From 2003 to 2005, the SFDA approved H101, experimental oncolytic viral therapy for head and neck cancer, an angiogenic Endostar inhibitor for treating non-small cell lung cancer and Gendicine for treating head and neck cancer [9–11]. However, in December, 2019, the Drug Administration Law (DAL) by the SFDA came into effect [12]. The DAL is likely to have an impact on experimental drugs because it addresses several issues such as public health concerns, drug innovation, drug safety and drug accessibility. In the “wake” of the recent epidemics such as Ebola, MERS caused by coronavirus (MERS-CoV) and more recently the outbreak of the coronavirus, SARS-CoV2 in 2019–2020, the use of “emergency drugs and compassionate use of experiential drugs” may warrant further considerations and the options available in an epidemic or a pandemic. Gilead Sciences’ experimental drug (at the time of writing), remdesivir, is one of the most promising drug candidates that may be effective against SARS-CoV2. The drug exhibits broad-spectrum antiviral activity against a number of RNA viruses including the Ebola virus by interfering with the viral polymerase enzyme. Remdesivir is currently being used as an “emergency experimental drug” on compassionate basis while randomized controlled studies are underway [13].

The risks and benefits of phase 1 oncology trials from 1991 to 2002 have been reviewed [14] involving a total of 460 trials and 11,935 participants. The participants were tested for toxicity and 10,402 participants were assessed for therapeutic efficacy. The overall response rate was reported to be 10.6% with considerable variations among trials. The classic phase 1 trials using single investigational chemotherapeutic drug represented 20% of the trials with a response rate of 4.4%. On the other hand, the trials that included at least one FDA-approved anticancer drug consisted of 46.3% of the trials and the response rate was 17.8%. The overall death due to toxicity was found to be 0.49%. The above study demonstrated the value and merits such as higher efficacy and safety due to a lower death rate if the investigational drug is combined with at least one FDA-approved anticancer drug. Some of the most recently phase 1/II completed studies (at the time of writing) are exemplified in Table 1 [15] and the drugs are potential experimental drug candidates.

**Table 1:** Example of anti-cancer drugs phase I/II completed studies

Disease or conditions	Interventions
Colorectal cancer	Drug: Capecitabine and Aflibercept
Metastatic breast cancer	Drug: Tivozanib (AV-951) + paclitaxel
Bladder cancer	Biological: Vesigenurtaclel-1 (HS-410) Biological: Placebo Biological: BCG
Ovarian cancer Primary peritoneal	Drug: Pemetrexed—Phase 1 Drug: Carboplatin—Phase 1 Drug: Pemetrexed—Phase 2 Drug: Carboplatin—Phase 2
Non-small cell lung cancer	Drug: Pazopanib Drug: Paclitaxel
Non small lung cancer	Drug: Pemetrexed Drug: Cisplatin Drug: Rabusertib (LY2603618)
Pancreatic cancer	Drug: Vantictumab (OMP-18RS) Drug: Nab-paclitaxel Drug: Gemcitabine

## Integrative synergistic approaches

Some of the most emerging and promising tools being developed and increasingly being used in the health care-related sectors include data warehouse, that is a repository of historical data from data warehouses to data refineries for refining the crude data dubbed as the “oil of the digital era” into valuable data all the way from research to clinical use [16, 17]. Because the data in the raw form is enormous, complex, lacks structure and standardization combined with interoperability issues, compliance issues and ethical challenges, the data refineries are envisioned to bridge the gap for refining and distilling the data on its journey from research to clinical utility for the benefits of the patients, including the stakeholders.

To navigate the costly and complex landscape of therapeutic drugs from basic research to clinical use hinges on integrating multi-disciplinary approaches. Because of different goals of the stakeholders, it has been historically challenging to strike a common chord that resonates across the whole ecosystem. Over the last few years, there has been a paradigm shift due to many factors such as the high cost of drug development, lengthy approval process, closer collaborations between academia and industries, integration of emerging technologies such as digital health, telehealth and wearables, gene editing, including big data, funding, education, and changes in government policies. The health benefits of panomics (genomics, proteomics, transcriptomics, metabolomics, epigenomics, ionomics and microbiomics) and the increasing use of panomics in personalizing medicine are emerging and promising in the treatment of diseases such as cancer, cardiovascular and gastrointestinal disorders [18, 19]. The integral role of “gut” microbiome in health and in treating many diseases, including cancer is beginning to emerge as demonstrated by immune checkpoint inhibitor therapy [20, 21]. While panomics addresses many of the precision medicine treatment benefits, it falls short in addressing issues such as the multi-morbidity, impact of the disease on the patients’ lives, their adaptability to the disease or other existing diseases, their family, their social life and their community life. Personomics is thus envisioned to bridge the gap between panomics and the patients’ personal or an individual’s circumstances [22]. This “echoes” with the words of Sir William Osler: “the good physician treats the disease; the great physician treats the patient who has the disease” [23].

The Precision Medicine Initiative (PMI) launched in 2015 [24] has been building to a “crescendo” and its impact on drug development, clinical trials and in personalizing the treatment for therapeutic efficacy, maximum safety, higher durable response, longevity and higher quality of life is emerging. Over the last few years, the FDA has emphasized the use of real-world data (RWD) and real-world evidence (RWE) to modernize clinical trials, an advancement made possible by the 21st Century Cures Act [25, 26]. With real-world data and real-evidence, researchers will be able to go beyond the scope of traditional trials, transition to a “hybrid” trial that is dynamic, providing insights from information collected in clinical care. As an example, the FDA in April 2019 approved a supplemental New Drug Application based on data extracted from EHR and post-marketing reports of the real-world use of Pfizer’s drug, IBRANCE (Palbociclib) to expand the indication for in combination with Fulvestrant to include men with hormone receptor positive (HR+), human EGFR 2 negative (HER2−) advanced or metastatic breast cancer, for the treatment of breast cancer in men [27, 28]. The former FDA Commissioner, Dr. Scott Gottlieb stated [29]: “the EHRs and other data sources, paired with advances in machine learning, will be crucial for architecting the next generation of successful clinical trials.

To address many of the challenges of implementing genomics medicine for routine use, the NIH funded IGNITE Network with the goals of integrating genomic data into EHR [30]. The IGNITE Network deploys plethoric “tools” for “Point-of-Care Decisions”, genetic markers for disease risk prediction including prevention, tools about family history data, pharmacogenomics data and refinement of disease diagnosis. Similarly, IBM Watson Health in collaboration with Brigham and Women Hospital and Vanderbilt University Medical Center has been pursuing the use of artificial intelligence for supporting precision medicine, to enhance patient safety, to nurture health equity, to expand and improve EHR usability [31]. Furthermore, the Watson Studio and Watson Knowledge Catalog has the data refinery tool for processing and transforming large amounts of raw data into valuable and clinical useful information for analytics. Several governments across the world, organizations, academia and institutes have created open access networks such as the Cancer Biomedical Informatics Grid (caBIG) and the Cancer Translational Research Informatics Grid (caTrip) for the caBIG project with a focus and a mission about driving translational research and improving the patient outcome by linking network of researchers, patients and physicians [32]. Similarly government and non-government sponsored programs have been established and they have been mushrooming globally such as the ICPerMed and the ECMC in the UK that support biotech and pharmaceutical companies to develop drugs in oncology through strategic partnerships [33]. Examples of programs in the USA include: the National Center for Advancing Translational Sciences (NCATS) at the National Institutes of Health (NIH), the NCI-MATCH, a precision medicine cancer treatment clinical trial that is co-led by National Cancer Institute (NCI) and the ECOG-ACRIN Cancer Research Group. In the NCI-MATCH trial, patients received therapy based on the genetic changes found in their tumor as exemplified by the results from Arm H of the study demonstrated that treatment with a “cocktail” of dabrafenib and trametinib, designed to target cancers that have specific BRAF gene mutations, was effective in a trial of 35 patients having 17 distinct tumor types [34]. Most recently, the studies [35] published by the Pan-cancer Analysis of Whole Genome (PCAWG) consortium involving whole genome sequencing of 2658 cancer genomes demonstrated new information about cancer drivers from 38 tumor types and identified potentially new targets for precision medicine.

The exploitation of such tools for the “Right-to-Try” experimental drugs in treating life threatening diseases such as cancer will most likely favor the outcome and mitigate the negative outcomes associated with the clinical use of experimental drugs. The outcome using experimental drug has the potential to be more favorable if combined (“cock-tail”) with an FDA-approved drug. Some of the other approaches include implementation of programs and policies that incorporate the interests of patients such as education, understanding of risks, second opinion about the therapy, expectations and costs due to potential complications, education of physicians about precision medicine and emerging tools, dosing, the use of EHR, systematic reporting of results that may be important for public health and safety. The sponsor should provide transparency and immediate notifications to the physician including the FDA about safety issues during the drug development stage and any manufacturing or supply issues. Due to the complexity of the informed consent form and lack of oversight by the FDA, it is important that an independent or a neutral body such as an IRB or an ethics committee is engaged in reviewing the consent procedures. The positive outcome of the treatment results will thus be a potential boon to young biotech and pharmaceutical companies facing the “valley of death” syndrome, struggling to raise funding or looking for partnerships or trying to build trust and credibility. Furthermore, a positive outcome has the potential to spawn new ventures or opportunities such as veterinary oncology and “outpatient” clinics.

It is envisioned that the use of new tools encompassing electronic medical records, personalized medicine, data refineries, artificial intelligence and machine learning, further testing of drugs, including adjuvant therapies or “cocktail” of drugs will favor the outcome of experimental drugs and may pave the way for indication expansion as exemplified by IBRANCE. Furthermore, the use of such tools are expected to: (i) accelerate drug development time; (ii) reduce drug development cost; (iii) lower the cost of drugs; (iv) improve the durable response; (v) reduce adverse effects such as hepato-and cardio-toxicity; (vi) improve longevity and quality of life of the patient.

## Conclusion

The progress made on several fronts in healthcare and the concerted efforts by the stakeholders, including the integral role of agencies such as World Health Organization (WHO) and Global Health Council (GHC) over the last few decades for the treatment of diseases, patient and public engagements, the role of healthcare practitioners, the role of education, data ownership, data sharing, transparency, privacy, ethics, standardization across the multi-industries, regulations, compliance, funding of programs, payment by medical insurance companies, including global policy development and implementation currently present limited opportunities and many challenges for the “Right-to-Try” experimental drugs for the treatment of life-threatening diseases. The “Right-to-Try” experimental drug is nevertheless a major “milestone” along the journey and its full impact on treating life-threatening diseases such as cancer and infectious diseases such as COVID-19 remain to be seen. One of the biggest impacts of emergency use of experimental drugs and compassionate drugs or “repurposing” of drugs is unfolding during the current coronavirus pandemic crisis.

## Data Availability

Data sharing is not applicable to this article because no data sets were generated and/or analyzed for the study.

## Abbreviations

ECMC: Experimental Cancer Medicine Center
EGFR: Epidermal growth factor receptor
EHR: Electronic health record
HER2: Human epidermal growth factor receptor 2
IRB: Institutional review board
MERS: Middle East respiratory syndrome
RNA: Ribonucleic acid
SFDA: State Food and Drug Administration (China)

## References

[1] Kantor AF (1976). Upton Sinclair and the Pure Food and Drugs Act of 1906: ‘I aimed at the public’s heart and by accident I hit it in the stomach. Am J Public Health.

[2] Jacobson PD, Parmet WE (2007). A new era of unapproved drugs: the case of Abigail Alliance v Von Eschenbach. JAMA.

[3] Mackey TK, Schoenfeld VJ (2016). Getting ‘social’ to access experimental and potentially life-saving treatment: an assessment of the policy and online patient advocacy environment for expanded access. BMC Med.

[4] https://www.abigail-alliance.org/story.php. Accessed 5 Dec 2019.

[5] Own D (2018). Trump signs bill to give patients right to try drugs. BMJ.

[6] Piel J (2016). Informed consent in right-to-try cases. J Am Acad Psychiatry Law.

[7] DiMasi JA, Grabowskib HG, Hansenc RW (2016). Innovation in the pharmaceutical industry: new estimates of R&D costs. J Health Econ.

[8] Mestre-Ferrandiz J, Sussex J, Towse A. The R&D cost of new medicine. 2012. https://www.ohe.org/publications/rd-cost-new-medicine. Accessed 7 Feb 2020.

[9] Tysome JR, Lemoine NR, Wang Y (2013). Update on oncolytic viral therapy—targeting angiogenesis. Onco Targets Ther.

[10] Jia H, Kling J (2006). China offers alternative gateway for experimental drugs. Nat Biotechnol.

[11] Zhang W, Li L, Li D, Liu J, Li X, Li W, Xu X, Zhang MJ, Chandler LA, Lin H, Hu A, Xu W, Lam DM (2018). The first approved gene therapy product for cancer Ad-p53 (gendicine): 12 years in the clinic. Hum Gene Ther..

[12] China’s New Drug Administration Law frames a promising regulatory landscape. 2019; 23:12. https://www.asiabiotech.com/23/2312/23120020x.html.

[13] https://www.gilead.com/purpose/advancing-global-health/covid-19. Accessed 13 May 2020.

[14] Horstmann E, McCabe M, Grochow L, Yamamoto S, Rubinstein L, Budd T, Shoemaker D, Emanuel E, Grady C (2005). Risks and benefits of phase 1 oncology trials, 1991 through 2002. N Engl J Med.

[15] https://clinicaltrials.gov/ct2/results?recrs=ab&cond=Lung+Cancer&term=&cntry=US&state=&city=&dist=. Accessed 7 Feb 2020.

[16] Boussadi A, Caruba T, Zapletal E, Sabatie BR, Durieux P, Patrice Degoulet P (2012). A clinical data warehouse-based process for refining medication orders alerts. J Am Med Inform Assoc.

[17] Using Watson Studio and Knowledge Catalog, Data Refinery saves data preparation time by quickly transforming large amounts of raw data into consumable quality information. https://www.ibm.com/cloud/data-refinery?p1=Search&p4=p50367843279&p5=b&p1=Search&p4=p50367843279&p5=b&p1=Search&p4=p50367843279&p5=b&cm_mmc=Search_Bing-_-1S_1S-_-WW_NA-_-%2Bdata%20%2Brefinery_b&cm_mmca7=71700000060884993&cm_mmca8=kwd-81226564052940:aud-807596473:loc-71283&cm_mmca9=CKT45PeiqOgCFQKrxQIdMe0EFA&cm_mmca10=81226482883052&cm_mmca11=b&&utm_source=bing&utm_medium=cpc&utm_campaign=Search%7CAI%7CLower%7CWW%7CNA%7CGeneric%7CEN%7CBMM&utm_term=%2Bdata%20%2Brefinery&utm_content=Data%20Refinery_Data%20Refinery_Generic_BMM_NULL&gclid=CKT45PeiqOgCFQKrxQIdMe0EFA&gclsrc=ds.

[18] Sandhu C, Qureshi A, Emili E (2018). Panomics for precision medicine. Trends Mol Med.

[19] Hasin Y, Seldin M, Lusis A (2017). Multi-omics approaches to disease. Genome Biol.

[20] Yi M, Yu S, Qin S, Liu Q, Xu H, Zhao W, Chu Q, Wu K (2018). Gut microbiome modulates efficacy of immune checkpoint inhibitors. J Hematol Oncol.

[21] Gopalakrishnan V, Helmink BA, Spencer CN, Reuben A, Wargo JA (2018). The influence of the gut microbiome on cancer, immunity, and cancer immunotherapy. Cancer Cell.

[22] Moor K, Heukels P, Kool M, Wijsenbeek M. Integrating patient perspectives into personalized medicine in idiopathic pulmonary fibrosis. https://www.researchgate.net/publication/321945065. Accessed 9 Jan 2020. 10.3389/fmed.2017.00226 PMC5742327 29326935

[23] American College of Physicians. “Sir William osler and internal medicine”. https://www.acponline.org/about-acp/about-internal-medicine/sir-william-osler-and-internal-medicine. Accessed 11 Jan 2020.

[24] Rothstein MA (2016). Some lingering concerns about the precision medicine initiative: currents in contemporary bioethics. J Law Med Ethics.

[25] Real-world evidence. FDA. http://www.fda.gov. Accessed 24 Mar 2020.

[26] 21^st^ Centuary Cures Act. https://www.fda.gov/regulatory-information/selected-amendments-fdc-act/21st-century-cures-act. Accessed 3 Feb 2020.

[27] Updated data from phase 3 trial of Ibrance (palbocicilib) plus letrozole in ER+, HER2− metastatic breast cancer confirm improvement in progression-free survival. https://www.pfizer.com/news/press-release/press-release-detail/updated_data_from_phase_3_trial_of_ibrance_palbociclib_plus_letrozole_in_er_her2_metastatic_breast_cancer_confirm_improvement_in_progression_free_survival. Accessed 8 Mar 2020.

[28] Taylor-Stokes G, Mitra D, Waller J, Gibson K, Milligan G, Iyer S (2019). Treatment patterns and clinical outcomes among patients receiving palbociclib in combination with an aromatase inhibitor or fulvestrant for HR+/HER2-negative advanced/metastatic breast cancer in real-world settings in the US: results from the IRIS study. Breast.

[29] Statement from FDA Commissioner Scott Gottlieb, M.D., on FDA’s new strategic framework to advance use of real-world evidence to support development of drugs and biologics. https://www.fda.gov/news-events/press-announcements/statement-fda-commissioner-scott-gottlieb-md-fdas-new-strategic-framework-advance-use-real-world. Accessed 8 Mar 2020.

[30] Implementing Genomics in Practice. https://gmkb.org/. Accessed 2 Mar 2020.

[31] David Raths. IBM Watson Teams with Vanderbilt, Brigham and women’s. Applying AI to improve utility EHR, claims data to address public health issues. 2019. https://www.hcinnovationgroup.com/analytics-ai/artifical-intelligence-machine-learning/news/21068437/ibm-watson-teams-with-vanderbilt-brigham-and-womens. Accessed 22 Feb 2020.

[32] McConnell P, Dash RC, Chilukuri R, Pietrobon R, Johnson K, Annechianrico R, Cuticchia JA (2008). The cancer translational research informatics platform. BMC Med Inform Decis Mak.

[33] https://www.ecmcnetwork.org.uk/. Accessed 4 Jan 2020.

[34] Jessica Kent. NIH precision medicine trial releases cancer study. 2019. https://healthitanalytics.com/news/nih-precision-medicine-trial-releases-cancer-study-results. Accessed 18 Jan 2020.

[35] Pan-cancer analysis of whole genomes. Nature. 2020;578:82–93. 10.1038/s41586-020-1969-6. 10.1038/s41586-020-1969-6 PMC7025898 32025007

